# A scoping study of cultural interventions to treat addictions in Indigenous populations: methods, strategies and insights from a Two-Eyed Seeing approach

**DOI:** 10.1186/s13011-015-0021-6

**Published:** 2015-07-04

**Authors:** Margo Rowan, Nancy Poole, Beverley Shea, David Mykota, Marwa Farag, Carol Hopkins, Laura Hall, Christopher Mushquash, Barbara Fornssler, Colleen Anne Dell

**Affiliations:** Department of Sociology, University of Saskatchewan, 1109 – 9 Campus Drive, Saskatoon, S7N 5A5 SK Canada; British Columbia Centre of Excellence for Women’s Health, E311-4500 Oak St, Box 48, Vancouver, V6H 3 N1 BC Canada; Bruyère Research Institute, 85 Primrose Avenue, Ottawa, ON K1R 6 M1 Canada; Department of Educational Psychology and Special Education, College of Education, University of Saskatchewan, 28 Campus Drive, Saskatoon, S7N 0X1 SK Canada; School of Public Health, University of Saskatchewan, 107 Wiggins Road, Room 2705, RUH, Saskatoon, S7N 5ES SK Canada; National Native Addictions Partnership Foundation Inc, Satellite Office 303 East River Road, Muncey, Ontario, N0L 1Y0 Canada; Department of Psychology and Northern Ontario School of Medicine, Lakehead University, 955 Oliver Rd., Thunder Bay, P7B 5E1 ON Canada

**Keywords:** First Nations, Cultural interventions, Addictions, Indigenous, Treatment interventions, Scoping study, Systematic review, Two-Eyed Seeing

## Abstract

**Background:**

This paper describes the methods, strategies and insights gained from a scoping study using a “Two-Eyed Seeing” approach. An evolving technique, Two-Eyed Seeing respects and integrates the strengths of Indigenous knowledge and Western sciences, often “weaving back and forth” between the two worldviews. The scoping study was used to inform a tool for measuring the impact of culturally based addictions treatment services on wellness in Indigenous populations. It formed part of a three-year study, *Honouring Our Strengths: Indigenous Culture as Intervention in Addictions Treatment*. The scoping study identified and mapped literature on cultural interventions in addictions treatment, and described the nature, extent and gaps in literature.

**Methods:**

Using a Two-Eyed Seeing approach, we adapted, applied and enhanced a common framework of scoping studies. In the end stage of the scoping review process, an Ad Hoc Review Group, led by our project Elder, reviewed and interpreted Indigenous and Western understandings within the mapped information. Elements of the scoping study were joined with results from community focus groups with staff at treatment centres.

**Results:**

Two-Eyed Seeing contributed differently at each stage of the scoping study. In early stages, it clarified team expertise and potential contributions. At the mid-point, it influenced our shift from a systematic to a scoping review. Near the end, it incorporated Western and Indigenous knowledge to interpret and synthesize evidence from multiple sources.

**Conclusions:**

This paper adds to the collective work on augmenting the methodology of scoping studies. Despite the challenges of a Two-Eyed Seeing approach, it enables researchers using scoping studies to develop knowledge that is better able to translate into meaningful findings for Indigenous communities.

## Background

This paper describes the methods, strategies and insights gained from bringing a “Two-Eyed Seeing” [1, 2] approach to a scoping study of the literature on Indigenous cultural interventions in addictions treatment. This research is part of a three year study, *Honouring Our Strengths: Indigenous Culture as Intervention in Addiction Treatment*, concerning the use of cultural interventions to support healing within addictions treatment of Indigenous people. The goal of the overall study was to develop an instrument to measure the impact of culturally based addictions treatment services on client wellness. The overall project uses several methods involving individuals with community-based wisdom to ensure that Indigenous ways of knowing are central to the study. The scoping study section was designed to identify publications on cultural interventions in the academic and grey literatures which could contribute to the overall study goal. A team of Indigenous and non-Indigenous researchers were committed to ensuring that Indigenous knowledge would be interpreted through a culturally based lens. The scoping study was not intended to validate knowledge generated from other methods, but rather to identify relevant literature and build an overall understanding of culture as an intervention in healing Indigenous people from addictions.

The scoping study team applied an Indigenous-centred guiding lens known as Two-Eyed Seeing in the course of gathering and assessing evidence. This approach has been adopted by the Canadian Institutes of Health Research (CIHR), Institute of Aboriginal Peoples’ Health [1]. Created by Mi’kmaq Elders Murdena and Albert Marshall, it was first used to develop an integrative science curriculum for post-secondary education [3], and subsequently applied to environmental sciences [4–7]. Martin [8] noted its potential value in better understanding the health issues facing Indigenous communities.

“Two-Eyed Seeing draws together the strengths of mainstream, or Western, and Mi’kmaq knowledges. The binocularity of this guiding principle means that by engaging the overlapping perspective of each ‘eye’, integrative science enjoys a wider, deeper, and more generative ‘field of view’.” ([2], p. 4–5) Two-Eyed Seeing is a “dance” between the Indigenous sciences’ “sense of the whole” for gaining understanding, and Western sciences’ “sense of the parts” [5]. It is a “weaving back and forth” between worldviews [3], with each “eye” alternating focus between its personal understanding and those of others in order to acquire new perspective, clarity and insight [8]. It allows the worldviews to remain autonomous, free from knowledge domination and assimilation. Two-Eyed Seeing was regarded by the late chief Charles Labrador of Acadia First Nation, Nova Scotia, as the roots of trees in a forest intertwining to hold hands [2].

Two-Eyed Seeing is evolving, opening opportunities for exploration and further definition. Its goal is to connect the best of Indigenous and Western knowledge systems, despite their fundamental differences in values and origins [9–11]. Indigenous knowledge derives from traditional teachings, empirical observations and revelations, and is conveyed through personal stories, holistic perspectives and metaphoric language [9]. Western academic knowledge in the social and health sciences has largely been rooted in positivist methods that privilege objective, linear, hierarchical, written evidence [10, 11].

Connecting these knowledge systems raises fundamental questions for research teams. The literature lacks guidelines on determining how to bring in the strengths from each knowledge system. In our project, Western and Indigenous processes were selected through negotiated and situated exercises. We consciously favoured Indigenous knowledge, historically muted or devalued by dominant Western thinking. That is not to say that we discounted Western knowledge; on the contrary, we applied Western-based methods as appropriate.

To our knowledge, a Two-Eyed Seeing approach has never been applied to a scoping study. We offer a new methodological advance of particular interest to those involved in Indigenous-centred research. The earliest scoping model, proposed by Arksey and O’Malley [12], had six stages:

1. Identifying the research question.
2. Identifying relevant studies found in published articles, papers or reports.
3. Study selection.
4. Charting the data.
5. Collating, summarizing and reporting the results.
6. Consultation with stakeholders (optional).

Other researchers have enhanced this model. Levac et al. [13] applied the framework in three scoping studies of different areas of primarily physical rehabilitation. Their recommendations clarified and enhanced each stage of the framework, such as using an iterative team approach to selecting studies (Stage 3) and requiring consultation with stakeholders as part of knowledge translation (Stage 6). Valaitis et al. [14] applied the first five stages of Arksey and O’Malley’s approach to a scoping review of collaboration between primary care and public health. They improved the process with various technologies, such as citation management software to organize the references (Stage 2) and qualitative data analysis software to compile and code information extracted from the literature (Stage 5). Daudt et al. [15] further enhanced the methodology by engaging an inter-professional team of 12. They identified the need to conduct scoping studies thoroughly and thoughtfully, in contrast to the original rapid design. They found that working with a large, inter-professional team added breadth and depth of knowledge.

In this article, we intend to add to the collective work on improvements and augmentations to scoping studies methodology. We note some key challenges with applying a Two-Eyed Seeing approach. Results of the scoping study have been reported elsewhere [16].

### Approach

Using a Two-Eyed Seeing approach, we adapted, applied and enhanced the six stage framework of Arksey and O’Malley [12] and Levac et al. [13]. We added a base stage to assemble an interdisciplinary, inter-professional and intercultural scoping study team [11].

The decision to switch to the scoping study was made at Stage 3, once we were made aware of the available literature to review. Our original intention was to conduct a Cochrane-style systematic review [17]. Systematic reviews have well-defined research questions, include a narrow range of quality assessed studies, have very specific inclusion/exclusion criteria, focus on randomized controlled trials (RCTs), assess for risk of bias and undertake meta-analyses to summarize studies. In contrast, scoping studies ask broad questions, can have *post hoc* inclusion/exclusion criteria, do not assess for bias or quality, and examine a wide range of evidence [13].

## Results and discussion

Table 1 summarizes the key milestones reached at each stage of the scoping study, and the contribution of Two-Eyed Seeing to each stage.

**Table 1:** Summary of Two-Eyed Seeing contribution at each stage in the scoping study

Scoping study stages	Key milestones reached	Two-Eyed seeing contribution
Base Stage: Creating a shared space for teamwork.	•Grounded selves in culture through active participation in ceremony.	•Exposed to different ways of knowing (Indigenous and Western) through immersion in cultural experiences and an understanding of team science.
	•Developed research principles for working together.	
		•Ensured the scoping team was balanced with a combination of Western and Indigenous thinkers, comfortable with the concept of spiritual wellness and able to create a shared space to converse and exchange knowledge.
	•Formed an interdisciplinary, inter-professional and intercultural scoping study of 11 team members.	
Stage #1: Identifying the research question.	•Research question established with an Indigenous lens: “What cultural interventions have been used to treat addictions in Indigenous populations and how effective are they?”	•Research question formulated through integrating Indigenous knowledge shared at initial full team meeting, with Western understanding of quality of evidence.
		•Guided by full team’s research principles—holistic research.
	•Supplementary objectives considered Western concepts of quality.	
Stage #2: Identifying relevant studies found in published articles, papers and reports.		•Applied an Indigenously-led perspective and Western-based vehicle to systematically search and screen the literature.
Stage #3: Study selection.	•Three rounds of relevancy testing.	•Influenced the switch from systematic review to scoping study to ensure openness to Indigenous context-dependent research as well as Western methods-controlled studies.
	•3,908 scientific articles and 610 grey literature reviewed.	
	•Final selection: 19 studies.	
Stage #4: Charting the data.	•Extraction form developed, piloted and applied.	•Used Western and Indigenous criteria to label and extract data.
Stage #5: Collating, summarizing and reporting.	•Narrative summaries and tables produced of descriptive and thematic information.	•Blended Western data and Indigenous knowledge.
Stage #6: Consultation with stakeholders.	•Meeting with Ad Hoc Review Group to further interpret and synthesize information.	•Synthesized Indigenous and Western knowledge from multiple lines of evidence to inform instrument development.

### Base stage: Creating a shared space for teamwork

Shared space must be created for dialogue between Indigenous and non-Indigenous partners involved in Two-Eyed Seeing approaches [9–11, 18–20]. Some see this space as a Venn-diagram with two partially overlapping circles, where Indigenous and Western knowledge intersect on common ground while maintaining their separate and individual identities [2, 8, 21]. Fornssler et al.’s [10] metaphor of a rhizome or growing root system encourages openness in thinking and connecting through differences and similarities. Others envision a space of ethical engagement forming “when two societies, with disparate worldviews, are posed to engage…and the space in between them…contributes to the development of a framework for dialogue between human communities” ([18], p. 193).

We created our shared space for the Two-Eyed Seeing approach in the scoping study by:

- Developing the research principles by which the *Honouring Our Strengths* project was intended to function,
- Grounding ourselves in culture through active participation in ceremony and
- Forming a large interdisciplinary, inter-professional and intercultural scoping study team similar to Daudt et al. [15].

#### Development of research principles

The full team first met in Saskatchewan from July 31 to August 2, 2012 to lay the foundation for the project. Most members of the research team participated, as did representatives from treatment centres and universities, policy-makers and community Elders. Western team research methods were presented, highlighting the science behind collaborative and cross-disciplinary approaches to generate and integrate knowledge [22, 23]. The literature on implementation of these approaches, particularly when working with Indigenous communities, is sparse [4, 10, 23]. Small group discussions among project team members at this meeting established our own set of 10 guiding research principles to shape the process and outcomes of Western and Indigenous knowledge generated in our work. (See Table 2.)

**Table 2:** Key guiding principles for the HOS-CasI project research process

1.	We are all in it together.
2.	Each person brings a part of the whole.
3.	Best use of talent and time – visibly and behind the scenes.
4.	Something for everyone.
5.	Using research to communicate and promote self-determination in the design and delivery of addictions and mental health by and for First Nations Peoples.
6	Holistic research – being open to unexpected outcomes.
7	Maintain momentum – keeping relationships over time.
8	Effective (multi-faceted) communication.
9	Make the best use of the research.
10	For the betterment of clients, we need to give the best of the best.

#### Grounding ourselves in culture

We extended the concept of team science – working collaboratively across disciplines [22, 23] – by building Indigenous ceremonial practices into our initial gathering. We listened to insights and teachings from Indigenous Elders on our project, incorporated smudging or burning sacred plants in prayer each day, and participated in a sweat lodge ceremony to begin our work. This is central to Indigenous ways of knowing and thus, our study. We consistently integrated the guidance of ceremony into all our gatherings. For example, we started and ended each meeting with a prayer in the language of the Indigenous speaker. Our team’s commitment to ceremony reflected our collective respect for the project’s focus and grounding in Indigenous knowledge and ways of knowing.

Incorporating traditional cultural ceremony meant different things to each member of our diverse team. For some, it was a way of connecting with the Creator to guide our team’s work together; for others, it was a new experience and much was learned. Team members participated in various ways, at various times and with various intentions throughout the research process. Some team members were First Nation and some were not so experiences varied. Grounding ourselves in traditional culture and ceremonies allowed our team to grow from a place of mutual respect, and to benefit from the guidance of Indigenous culture.

#### Formation of the scoping study team

Forming an interdisciplinary, inter-professional and intercultural scoping study team was key to creating a shared space for teamwork. As in other scoping studies [13], teams share expertise and increase efficiency in managing the extensive information under review [15]. Our scoping team included 11 members of the full project team, including Indigenous and non-Indigenous research scientists of varied backgrounds: First Nations Mental Health and Addictions, Sociology, Psychology, Education, Evaluation, Epidemiology, Women’s Health and Public Health. All expressed particular interest in the review and/or had expertise in various Western science review methods. Some members were also on the Indigenous Knowledge team, the designated lead on instrument development. This team comprised our project Elder Jim Dumont, a co-Principal Investigator, an Indigenous researcher, and two Indigenous staff and Knowledge Keepers from treatment centres. Importantly, team members were comfortable discussing issues inherent in Indigenous well-being – particularly the spirituality dimension of health that Western science often fails to address [11].

The mutual respect of Indigenous and non-Indigenous team members facilitated open, rich conversations and genuine engagement. It also allowed us to “always be looking for another perspective” ([3], p. 336) with the goal of linking (not assimilating) knowledge systems, exploring differences, and naming and questioning assumptions [24]. Team members were necessarily flexible and adaptive. Indigenous scientists were invited to apply Western methods to systematically extract information from studies, or to apply Indigenous knowledge to categorize cultural interventions. Western scientists readily stepped back from their knowledge base to welcome the wisdom of Indigenous thinking throughout the scoping study.

### Stage 1: Identifying the research question

This stage was guided by the original project intent: To create a valid, reliable, culturally competent instrument (with careful attention to group process) for measuring the effectiveness of First Nations culture as an intervention in the treatment of alcohol and other drug addictions. Effectiveness was inferred by evaluating client change during and after participation in the treatment program. The research question was further developed to maximize coverage in identified articles and reports [13]: What cultural interventions have been used to treat addictions in Indigenous populations, and how beneficial are they?

A Two-Eyed Seeing process was used at this stage, integrating Western and Indigenous knowledge in formulating the research question. Its Western aspect focused on assessing the quality of studies in terms of design and measurement. On the Indigenous side, we were guided by what we experienced and heard during our initial full team meeting. We were particularly influenced by information from the project’s Foundation paper [25], which introduced and outlined key concepts about the qualities of cultural interventions within an Indigenous frame of reference. The research question was broad, in keeping with our research principle of holistic research. This approach suggested that the outcomes of our research could be widely applicable, from mainstream thinking to cultural programming, and that qualitative (narrative or interpretive) data were of equal importance to quantitative (variable-analytic and statistical) data. Unexpected outcomes were welcome.

### Stage 2: Identifying relevant studies

Three elements of the “PICO” (Patient/population, Intervention, Comparison, Outcome) method [26] were used to identify studies in articles, papers or reports from published literature. Population included First Nations, Indigenous and Aboriginal people in treatment for problematic substance use or addictions. Cultural interventions were defined as Indigenous spiritual and healing practices or traditions introduced to residential or outpatient treatment centres, with the goal of achieving wellness in recovery from addiction. These initially included those listed in the Foundation paper, such as sweat lodge ceremonies and traditional teachings [25]. We did not require studies that made comparisons between treatment and control groups. The four dimensions of Indigenous wellness listed in the Foundation paper [25] were searched under “Outcomes”:

- Spiritual
- Heart/Social/Emotional
- Mind/Mental and
- Physical/Behavioural

The PICO method [26] is a Western science tool used in conducting searches for knowledge synthesis, particularly systematic reviews. We used it to determine the relevance of studies. A spreadsheet of titles, exclusion criteria applied at various levels of screening, and areas of agreement and disagreement, yielded transparency and reproducibility. However, PICO did not drive the search. With the Two-Eyed Seeing approach, we used search terms and parameters from an Indigenous perspective. This was grounded in the Foundation paper [25], which discusses wellness as involving the whole person – spirit, heart, mind and body. It gives examples of cultural interventions including sweat lodge, fasting ceremony, natural foods and medicines, singing and dance. We relied on this paper and further insight from its lead author to identify relevant cultural interventions and holistic outcomes associated with the four dimensions of Indigenous wellness. This definition was enormously valuable to the expert librarian on the team who would otherwise have used common Western parameters that focus on physical health, specifically reduction in alcohol or substance use.

A library scientist worked with the project team to develop the PICO criteria and run the search. Another librarian peer-reviewed the search strategy using PRESS [27]. The final strategy was first developed in MEDLINE and then applied to the other databases. A total of 25 databases were searched, 12 of scientific literature and 13 of grey literature (Table 3), ending October 26, 2012. We also sought articles in other ways, such as searching reference lists of included studies. The search identified studies of all languages that involved primary data collection, literature and systematic reviews, background information about culture as an intervention or wellness as a concept, and instruments and measures.

**Table 3:** Electronic databases searched for scoping study

Scientific Literature	Grey Literature
•EBM Reviews (including The Cochrane Library)	•Google 1, Google 2, Google Scholar
	•North American Indian Thought and Culture Database
•Global health library	
	•ARTIS (Arctic Science and Technical Information System)
•MEDLINE	
	•Arctic Health
	•First Nations Behavioral Health Associations
	•One Sky Centre
•EMBASE	
•PsycINFO	
•Bibliography of Native North Americans	
•CINAHL	
•Social Work Abstracts	•US Indian Health Service
	•American Society of Addictive Medicine
	•National Indian Health Board
•Women’s Studies International	•Native Health
	•CDC (US Centers for Disease Control and Prevention)
•Anthropology Plus	
•Anthropological Literature	
•Anthropological Index	
•CAB direct	

Although we anticipated that the original list would be expanded, it provided a solid launch to the process. A purely Western perspective would likely have produced a fairly generic set of search parameters that may have missed relevant articles and reports. Yet Western search techniques allowed us rapid access to a wealth of national and international literature that greatly enhanced our understanding of Indigenous wellness. The weaving between Western and Indigenous knowledge systems allowed us to select pieces of either origin at different times during the search process, to the overall benefit of the scoping study.

### Stage 3: Study selection

Studies were evaluated using a three-phase method involving five reviewers of the grey literature (DM, MF, NH, RD, RU), eight reviewers of the scientific literature (BF, BS, CD, CH, JPG, LH, MR, NP), and one main arbiter (MR). At least one First Nation team member was involved in each phase. In the first phase of screening, two independent reviewers assessed the title and abstract of each scientific article (MM, MR) or report (RD, RU) against our PICO criteria. The full article was scanned when relevance was unclear from the abstract. Disagreements were resolved by consensus.

In the second phase, separate reviewers (BF, BS, CD, CH, DM, JPG, LH, MF, MR, NH, NP, RD, RU) applied the same criteria to the full articles or reports selected in phase one. If it was unclear whether an article or report should be excluded, the arbiter and reviewer discussed it further and came to an agreement. The third phase of review was during extraction. Fifty-six articles were reviewed in detail and excluded at this point. The process was similar to phase two. A total of 19 English language articles were included: 14 from the scientific literature and five from the grey literature.

Building a strong foundation of knowledge to inform the creation of the instrument required an in-depth understanding of the literature using a Western science knowledge synthesis method. There are dozens of approaches to synthesize and interpret literature [28, 29], and we had initially decided that a systematic review would be the most suitable. It is well understood and adopted in the scientific community, and is rigorous in aggregating, summarizing and synthesizing research findings in support of policy decisions [29, 30].

The Two-Eyed Seeing approach was instrumental in leading us from a systematic review to a scoping study. At our first team gathering, several members voiced serious reservations about how a systematic review fit with our study. These reviews privilege RCTs and thus limit inclusion of other, context-dependent forms of evidence [29, 31]. A systematic review training session reinforced the emphasis on controlled studies. Only two RCTs were found out of about 2300 abstracts considered. We had to make an important decision about our literature review method.

A scoping study would be more relevant to the Indigenous worldview in our work, yet the systematic review was the approach put forward in the project proposal. The tension between these two methods led to a decisive shift to prioritizing relevant information. Such tensions in projects that address both Western and Indigenous knowledge are recognized as productive, encouraging teams to consider what is best for their project [10]. To engage in the Two-Eyed Seeing process, we needed to “learn to co-learn” ([6], p. 75). We redirected our approach to open the team to different forms of evidence while respecting our established research principle of conducting a holistic inquiry. We chose to explore the research and consider emerging insights from an open range of study designs: quantitative, qualitative and mixed method, and from both scientific and grey literatures.

After considering the nature of the literature, the group decided that a scoping study would be the appropriate option. The transition was simple at this point, as the first two stages of the systematic review and scoping study are similar. The scoping study proved a good match with our research objectives. Our decision was inspired by the need to select a review method that would best inform our Indigenously-centred research project [8]. Compared to a systematic review, a scoping study is less focused on control, reductionism and rigorous analysis. It identified information that is connected to culture, accessible, relevant and helpful in learning and understanding the effects of culture on addiction treatment among Indigenous people. The scoping study allowed us to include different types of studies, providing wider evidence to contribute to and integrate with Indigenous knowledge in instrument development. We were able to include articles about culturally based approaches whose elements might be delivered during treatment. Similar to the Indigenous organic process of “knowledge gardening” [5, 32], what the scoping study may have lacked in depth and rigor, it gained in breadth and relevance.

### Stage 4: Charting the data

Nine research team members charted the data. Information was extracted from articles using a customized and pilot-tested form (available on request). The form summarized the purpose of each study, its design, participants, context, type of study (prevention or treatment), outcomes (in four areas of wellness), measurement (quantitative or qualitative), results, appropriateness of the research from an Indigenous perspective, and significance of gender. As suggested by Levac et al. [13], the form was piloted by two researchers (MR, RD) who assessed its consistency with the research question and purpose, completeness and ease of use. On review of the seventh article, no new changes were made to the extraction form and piloting ceased. The charted information was entered into Microsoft Word files and shared with the entire research team. Additional summary tables outlined reasons for excluding articles at each phase of assessment.

Using a Two-Eyed Seeing approach, the extraction form combined Western and Indigenous evaluation criteria. Western criteria were used to label and extract types of study design (experimental, quasi-experimental, mixed methods, qualitative and other) and outcomes (significance, validity and reliability of tools used). We collected detailed information about the types of cultural interventions which had been categorized using labels and descriptions from the Foundation paper [25]. We relied on Indigenous meanings of wellness under the four main themes (Spiritual, Heart/Social/Emotional, Mind/Mental, Physical/Behavioural) [25]. In accordance with Daudt et al’s [15] assertion that assessment of study quality is a necessary part of scoping studies, supplementary information was charted to evaluate study design and data collection instruments. Finally, we collected information about the appropriateness of the research process of each study from an Indigenous perspective, with a particular focus on adherence to the principles of ownership, control, access and possession of data (OCAP) [33].

### Stage 5: Collating, summarizing and reporting the results

A descriptive-analytical method was used to collate and summarize the information [12]. Analytical frameworks were applied to all articles and reports, and relevant information on each study collected. Charted information was synthesized, tabulated and summarized to identify trends and themes relevant to the research question. Basic metrics of the characteristics of interventions and studies, and thematic analyses on wellness outcomes, were noted. Subthemes with common meanings were then created and described.

Presentation of results accommodated and integrated both worldviews of the Two-Eyed Seeing approach. One table detailed each program’s cultural interventions (e.g. ceremonial practice) and Western-based practices (e.g. individual and group counselling). Another table took a decidedly common Western approach, objectively describing the samples, design and method of each study, enabling critical assessment of their quality. Finally, results of each study were categorized in the four main areas of Indigenous wellness. This method was consistent with published discussions of Two-Eyed Seeing. For example, as a model for “co-advancement” ([11], p. 3), Two-Eyed Seeing tries to avoid knowledge dominance – the hierarchical approach to knowledge construction usually led by science [4]. Two-Eyed Seeing invites the views of both worlds to combine the knowledge, skills and values of Indigenous ways of knowing and Western science [2, 4]. There is no attempt to balance each worldview, as the weaving back and forth selects the knowledge system with the more applicable strength in changing circumstances [3].

### Stage 6: Consultation with stakeholders

Although optional to Arksey and O’Malley [12], we found – as did Levac et al. [13] – that consultation with stakeholders was essential. Over and above consultation, our process created and maintained respectful relationships, and incorporated multiple Western and Indigenous perspectives in keeping with our Two-Eyed Seeing frame of reference. The scoping study provided valuable information from national and international literatures. We were able to determine what others had found about, for example, various types of cultural interventions, and how Indigenous wellness themes and subthemes were measured. Our primary method of gathering Indigenous knowledge was focus groups with 12 National Native Alcohol and Drug Abuse Program (NNADAP) and Youth Solvent Addiction Program (YSAP) treatment centres participating in the project. NVIVO™ 9 qualitative analysis software was used to code and summarize the information collected in the focus groups. Our project Elder spoke with each treatment centre about the meaning of Indigenous wellness and how it is achieved in their treatment programs. The Indigenous Knowledge Team consulted further with all treatment centres to confirm the major themes arising from focus groups to develop an Indigenous Wellness Framework.

Information from Indigenous focus groups and the Western scoping study were mapped in tables to highlight major areas of overlap and gaps in identified cultural interventions and wellness outcomes. An Ad Hoc Review Group reviewed and interpreted Indigenous and Western understandings of the mapped information. This Group comprised Indigenous leaders, including our project Elder, Principal Investigators, those collecting and analysing the results from the focus groups, and members of the scoping study team. The group met in Alberta, Canada on July 20 and 21, 2013. Using Indigenous information as the primary base, we assessed similarities and differences with information from Western sources, and whether any differences should be accounted for in the Wellness Framework. By exploring relationships and links across the evidence, we combined descriptions of separate findings into a meaningful identification of commonalities and a discovery of differences from Indigenous and Western sources. For example, the literature identified 17 cultural interventions. Although there was overlap, our community of treatment centres identified several additional interventions with regional variation across Canada. Results were reported to the project’s National Gathering, where treatment centres and researchers shared findings, validated information and developed indicators of wellness.

### Challenges with applying the two-eyed seeing approach

This paper focuses on the benefits of applying the Two-Eyed Seeing approach in our study, in keeping with the strengths-based foundation of our study. However, application of the Two-Eyed Seeing approach was not without challenges. In the Base Stage, members of our team grounded themselves in Indigenous culture through active participation in ceremony. Individuals’ interpretation of “active” varied. Indigenous peoples and cultures are foundational to the formation of Canada, and our country has a colonial history. Team members had been educated differently about our history, leading to unintended interpersonal tensions. These and other historical disconnects are not easy to address; their contemporary ramifications cannot be overlooked.

## Conclusion

A Two-Eyed Seeing approach that acknowledges and accepts different ways of knowing in the Indigenous and Western worldviews was particularly helpful to our scoping study. It effectively addressed the heterogeneous nature of the literature and the need to include diverse research designs and methods. Our team’s comfort and ability to apply a Two-Eyed Seeing approach evolved throughout the study. We realized the benefits of learning to see from one eye with the strengths of Indigenous knowledge, and from the other eye with the strengths of Western knowledge [3], and to switch our focus in response to changing needs within the study. Two-Eyed Seeing opens new opportunities for researchers using scoping studies to uncover and develop knowledge that can translate into meaningful findings for Indigenous communities.
